# Living arrangements and depression of the older adults– evidence from the Chinese longitudinal healthy longevity survey

**DOI:** 10.1186/s12889-023-16730-4

**Published:** 2023-09-27

**Authors:** Qingwen Jia, Yanhan Duan, Rui Gong, Meijun Jiang, Dianping You, Yi Qu

**Affiliations:** 1 Organization and Personnel Department, Children’s Hospital of Hebei Province, Shijiazhuang, China; 2 Medicine-Education Coordinateion and Medical Education Research Center, Hebei Medical University, Shijiazhuang, China; 3 Department of Adult Internal Medicine, Children’s Hospital of Hebei Province, Shijiazhuang, China; 4 Graduate School, Hebei Medical University, Shijiazhuang, China; 5 Party and Government Integrated Office, Children’s Hospital of Hebei Province, Shijiazhuang, China; 6 Scientific Research Division, Children’s Hospital of Hebei Province, 133 Jianhua Street, Yuhua District, Shijiazhuang, Hebei China

**Keywords:** Depression, Older adults, Living arrangements, Living arrangement preferences

## Abstract

**Background:**

The mental health and living arrangements of older adults are worthy of attention. Previous studies have pointed out that the living arrangements may be related to older adults’ depression. However, it has not been found that studies concern the relationship between actual living arrangements, living arrangement preferences, and the fit between living arrangement preferences and reality and depression in older adults, so we carried out this study.

**Methods:**

The data from the Chinese longitudinal healthy longevity survey were used in this study. With the older adults’ depression as the dependent variable and the living arrangement related variables as the independent variable, we constructed three binary-logistic regression analysis models to explore the potential relationship between living arrangement related variables and depression in older adults.

**Results:**

We found that the actual living arrangements, living arrangement preferences, and the fit between living arrangement preferences and reality are significantly correlated with depression in older adults. Specifically, older adults living alone or only with the spouse are at greater risk of depression. Older adults who prefer living alone or only with the spouse are at relatively low risk of depression. Older adults whose living arrangement preferences do not match reality have a higher risk of depression.

**Conclusion:**

The living arrangement related variables are significantly correlated with depression in older adults. In addition to the actual living arrangements, living arrangement preferences and whether the living arrangement preferences fit with reality are also related to the depression of older adults.

**Supplementary Information:**

The online version contains supplementary material available at 10.1186/s12889-023-16730-4.

## Introduction

With the progress of medical treatment, society, and the economy, population aging has gradually become a trend. In 1960, the world's resident population was 3033 million, of which 5% were older adults 65 years old and above [1]. In 2022, the world population will reach 79.54 billion, of which older adults aged 65 and over will account for 10% of the total population, and even 20% in more developed regions [2]. It is estimated that by the middle of this century, older adults aged 65 and over will account for 16% of the total population in the world, while in more developed regions, this proportion may rise to 27% [3]. Facing the wave of population aging, China is also facing very serious challenges due to its huge population base. According to previous data, there were 190 million people aged 65 and over in China, 13.50% of the total population [4].

With the process of population aging, facing the growing demand for elderly care and related challenges, elderly care, and elderly health have increasingly become one of the focuses of attention in many countries. Earlier, the World Health Organization (WHO) came up with the development concept of “active aging”, pointing out that "Active aging is the process of optimizing opportunities for health, participation and security in order to enhance quality of life as people age" [5]. Subsequently, the National Population Development Plan (2016–2030) was issued by the State Council, which sets the direction for the construction of China's elderly care system. It proposed to implement "active aging" and give priority to the elderly care related construction, which means that the development concept of "active aging" is combined with China's actual situation [6, 7].

In Chinese society, caring for older adults is usually considered a family responsibility, not a social problem [8]. Under the influence of traditional ideas, the older adults who need to be cared for are mainly taken care of by their families or relatives, while other services such as public welfare or market-oriented services are only supplementary [9, 10]. Although it has always been one of the most important living arrangements for older adults in China to live with children [11], with the increase of the aging population, empty-nest families, and floating population, most young people are unable to perform filial piety [12]. According to the seventh population census, China's average household population has less than three for the first time in 2021. The shrinking size of families makes the problem of empty nests increasingly serious [13]. This means that not living with children is also becoming one of the important living arrangements for older adults.

The health of older adults should be highly valued, because aging will lead to many changes, affecting various parts of the body, such as the immune system, cardiovascular system, etc. Many changes caused by aging affect the components of the immune system, which is called "immune aging", further leading to the increase in the incidence rate and mortality of elderly organisms caused by infectious diseases [14, 15]. In contrast, older people seem to be more susceptible to cardiovascular disease, because aging can damage the function of the cardiovascular system [16]. According to the previous statistics of the American Heart Association, the average incidence rate of cardiovascular diseases in people aged 60–80 years is 75–78%, and this proportion even exceeds 85% among people aged 80 years and above [17].

Health is not just about physical fitness. Because health is considered "a state of complete physical, mental and social well-being and not merely the absence of disease or infirmity", which means that mental health is also an integral component of health [18, 19]. Based on the data from the World Health Organization, the most common neuropsychiatric disorders among adults aged 60 years and above are depression and dementia [20]. When adults enter old age, they are often accompanied by changes in family roles and social roles, as well as a decline in physical functions. If older adults can’t treat these changes rationally, it may lead to depression [21]. Depression has been proven to cause many adverse consequences, such as decreased subjective cognitive ability, general dysfunction, and suicide [22–26]. In China, depression is one of the top ten causes that affect disability adjusted life expectancy (DALY) [27], and it also brings a huge burden of disease. It is estimated that by 2030, the disease burden of depression is expected to exceed that of all other diseases [28]. Therefore, it is necessary to be concerned about older adults’ depression.

At present, many researchers are focusing on the depression of older adults. They found that intergenerational relationships, family social support, and social participation are significantly correlated with depression among older adults [21, 29]. Some researchers have also paid attention to the influence of older adults’ living arrangements on their mental health. Xu et al. found that there was a significant relationship between the actual living arrangements and older adults’ depression in China. Older adults living alone or in elderly care institutions have a higher risk of depression, compared with older adults living with their families [30]. A large sample study from South Korea also confirmed that living alone is an independent risk factor for depression in older adults. When other confounding factors were controlled, the risk of depression in older adults living alone was 1.45 times higher than that in older adults not living alone [31]. However, these studies only considered the potential relationship between the actual living arrangements and depression, while the possible role of living arrangement preferences was not considered. Based on previous studies, Chen found that the fit between living arrangement preferences and reality can be negatively related to the depression of older adults, and after adding this variable, the relationship between actual living arrangements and depression is no longer statistically significant. Therefore, he emphasized that the preferences of older adults should be carefully considered in the research process of exploring the relationship between living arrangements and depression [32]. However, we have not found any research focusing on the potential association between living arrangement preferences and depression. To further confirm the relationship between living arrangement preferences and older adults’ depression, and supplement the literature in this field, we carried out this study.

## Material and method

### Data source

The data comes from the Chinese Longitudinal Healthy Longevity Survey (CLHLS) conducted by the Center for Healthy Aging and Development Studies, a follow-up survey on older adults, covering 23 provinces, cities, and autonomous regions. The whole survey includes two parts: the survey for the survivors and the survey for the relatives of the elderly who died. We focus on the relevant data of the survivors, and the questionnaire includes basic information, health status, living arrangements, and other contents. Since the implementation of CLHLS in 1998, the survey project has carried out eight waves of tracking surveys, the most recent tracking survey was in 2017–2018. The Biomedical Ethics Committee of Peking University approved CLHLS (IRB0000105213074). All participants expressed written informed consent. All data of CLHLS are publicly available [33].

**Fig. 1:**
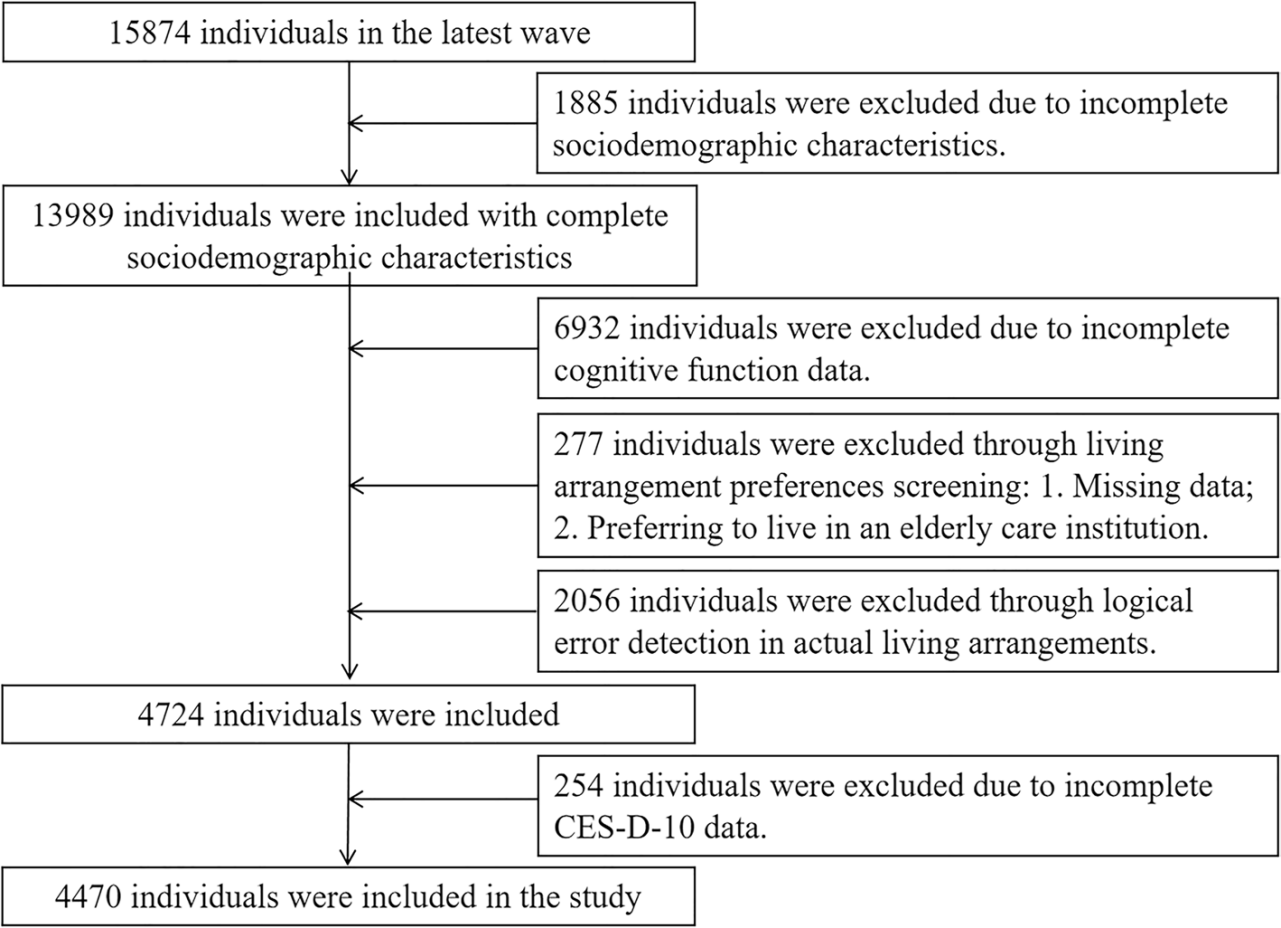
Flowchart of participant selection

We included CLHLS participants aged 65 and above in the latest survey wave (2017/2018) in our analysis to reflect the latest status of social relations among older adults in China. The latest wave includes 15874 individuals. First, we excluded 1885 individuals with incomplete information on social demography characteristics, including lack of information on age, marriage, economic status, perceived health, and perceived health changes. Subsequently, 6932 participants were excluded due to incomplete data on cognitive function. Then 277 individuals were excluded based on living arrangement preferences due to missing information or preferring to live in the institution. Subsequently, the actual living arrangement data was screened, and a comprehensive logical check was conducted using the three variables provided in the questionnaire, namely the actual living arrangements, the number of co-residents, and the relationship with the interviewee: a. Exclude actual residence in institutions or incomplete data; b. Due to the limitations of the questionnaire, data with more than 10 co-residents are excluded; c. Using actual living arrangements, number of co-residents, and the relationship with the interviewee for logical error checking: If a person is actually living alone, there should be no data on the number of co-residents and the relationship with interviewee; If a person actually lives with their family, when the number of co-residents is 1, the first record in the relationship with interviewee should have data, and the following nine records should be blank. If a person actually lives with their family, when the number of co-residents is 2, the first two records in the relationship with interviewee should have data, and the following eight records should be blank. If a person actually lives with their family, when the number of co-residents is 3, the first three records in the relationship with interviewee should have data, and the following seven records should be blank. If a person actually lives with their family, when the number of co-residents is 4, the first four records in the relationship with interviewee should have data, and the following six records should be blank. If a person actually lives with their family, when the number of co-residents is 5, the first five records in the relationship with interviewee should have data, and the following five records should be blank. If a person actually lives with their family, when the number of co-residents is 6, the first six records in the relationship with interviewee should have data, and the following four records should be blank. If a person actually lives with their family, when the number of co-residents is 7, the first seven records in the relationship with interviewee should have data, and the following three records should be blank. If a person actually lives with their family, when the number of co-residents is 8, the first eight records in the relationship with interviewee should have data, and the following two records should be blank. If a person actually lives with their family, when the number of co-residents is 9, the first nine records in the relationship with interviewee should have data, and the following one record should be blank. If a person actually lives with their family, when the number of co-residents is 10, there should be data in the top ten records of the relationship with the respondent, 2056 individuals were excluded through this step. Finally, 254 individuals were excluded due to incomplete CES-D-10 data, and 4470 individuals were retained. Refer to Fig. 1 for a specific flow chart.

### Determination of variables

#### Dependent variable (Depression)

In this study, we took the depression of the respondents as the dependent variable. The depression was obtained through the 10-item Center for Epidemiologic Studies Depression Scale (CES-D-10), which is often used to assess older adults’ depression in China, with good reliability and validity [34, 35]. CES-D-10 has ten items in total, and the score of each item is divided into four grades: 0 = "rarely" to 3 = "most of the time". For two of the positive questions, "I was happy" and "I felt hopeful about the future", the score was inversely encoded before summing. The maximum CES-D-10 score is 30, and the higher the score, the higher the severity of depression. If a person scores no less than 10 points in CES-D-10, he/she is considered to have depressive symptoms. This critical value can be found in many previous studies and has been fully verified in the depression measurement of older adults in China [35, 36]. The Cronbach's alpha of CES-D-10 in this study is 0.710, which means that the internal consistency level is reasonable.

#### Independent variable (Living arrangements)

We take the variables related to older adults' living arrangements as the independent variables of this study, including preference, reality, and the fit between preference and reality.

First, older adults' living arrangement preferences are extracted from the questionnaire's personal background and family structure. Choose the question "Which kind of living arrangements do you prefer" to measure the respondents' real preferences. This question can only be answered by older adults themselves, so we think it can reflect the real willingness of the interviewees. This question contains four options: a. Live alone or with the spouse, no matter how far your child lives; b. Live alone or with the spouse, but preferably with children living nearby; c. Live with children; d. Institutions (senior centers, nursing homes, etc.). Since this study does not focus on those respondents who choose elderly care institutions, we exclude these respondents. Then, we combined the options "a" and "b", coded them as living alone or only with the spouse, and finally formed two living arrangement preferences: a. living alone or only with the spouse and b. with children.

The second is the actual living arrangements, which are taken from the basic information part of the questionnaire. Similar to the living arrangement preferences, the actual living arrangements are also divided into institutions, with household members and living alone. Similarly, since we do not pay attention to respondents who choose elderly care institutions, we exclude these respondents. Then we logically divide the actual living arrangements into a. living alone or only with the spouse, and b. living with children, based on the actual living arrangements, the number of co-residents, and the relationship with the interviewee.

The last is the fit between living arrangement preferences and reality. As an important independent variable, we take older adults’ living arrangement preferences as the measurement standard and compare the actual living arrangements and preferences of the respondents to determine whether they fit.

#### Covariates

We included the following variables as covariates: gender, age, marital status, residence, economic status, perceived health, perceived changes in health compared to a year ago, and cognitive function to control for confounding factors. These covariates have also been used as potential confounders in previous studies [30, 37–39]. For marriage, the original data includes five parts (married and living with the spouse; married but not living with the spouse; divorced; widowed; never married). Two options are finally formed after the combination of options: a. married and living with the spouse and b. others. For the economic situation, we use "How do you rate your economic status compared with others in your local area?" to ask the interviewees to judge their economic level in the local area. This question contains five options: 1 = very rich to 5 = very poor. Finally, we combine the interviewees into 1 = rich to 3 = poor. The evaluation of the health-related status of older adults is based on subjective questions to obtain information, including self-perceived health and changes in health compared to last year. Both of these questions used a 5-point scoring system, and in the end, we merged the options to form a new code, which is 1 = good to 3 = bad, and 1 = better to 3 = worse. For the cognitive function of older adults, the Chinese version of the Mini-Mental State Examination (CMMSE) was used for evaluation, adapted from the scale originally developed by Folstein et al. [40]. CMMSE has been validated in older adults in China [41], with a score range of 0 to 30. The higher the CMMSE score, the better cognitive function. According to previous research recommendations [42], in this study, a CMMSE score below 18 is considered a cognitive impairment, while a score greater than or equal to 18 is defined as normal.

### Statistical analysis

First, descriptive analysis was used to describe the socio-demographic characteristics of participants, and the chi-square test was used to evaluate the difference in depression among various socio-demographic variables. Then, the potential relationship between each variable and depression was explored using binary logistic regression analysis. Through the binary logistic regression model, we first explored the potential relationship between socio-demographic characteristics and actual living arrangements and depression in model 1. Later, we further added variables to model 1, including living arrangement preferences and the fit between living arrangement preferences and reality, to form models 2 and 3, to probe the potential association between these variables and depression. Finally, the corresponding odds ratio (OR) and 95% confidence interval (95% CI) were reported. All data were analyzed by SPSS 22.0, with a statistical significance of bilateral *p* < 0.05.

## Result

### Participant characteristics

After data screening, 4470 respondents were finally included in this study. Figure 1 shows the specific process of screening. Among them, males account for 53.15%. The average age of the respondents is 80.09 ± 9.50 years old. The respondents mainly came from rural (41.39%), and the respondents from cities accounted for 28.25%; The majority of respondents (69.30%) reported that their economic status was common, and 7.99% of the respondents thought that their economic status was poor. For health, 52.44% of older adults feel that they are healthy, while 36.78% of older adults feel that their health is average. Compared to last year's health status, 53.26% of older adults feel that there has been no change, while 32.24% of older adults feel that their health has deteriorated. In terms of cognitive function, the majority of older adults have normal cognitive function (99.04%), and only 0.96% of older adults have cognitive impairment. In the actual living arrangements, the majority are living alone or only with the spouse (67.74%), and only 32.26% are with children. Similarly, in terms of living arrangement preferences, the majority of older adults prefer to live alone or only with their spouse (64.83%), while 35.17% prefer to live with their children. When comparing the living arrangement preferences with reality, 84.34% said they met their willingness. In addition, when using the chi-square test for univariate analysis, it was found that depression differs in sociodemographic variables such as gender, residence, and marital status (See Table 1).

**Table 1:** Sample characteristics and univariate analysis of depression

Variables		N (%)	Depression		Chi-square	*P*	Cramer's V
			No (78.88%)	Yes (21.12%)			
Gender	Male	2376 (53.15%)	1954 (82.24%)	422 (17.76%)	34.324	< 0.001	0.088
	Female	2094 (46.85%)	1572 (75.07%)	522 (24.93%)			
Residence	City	1263 (28.25%)	1034 (81.87%)	229 (18.13%)	15.639	< 0.001	0.059
	Town	1357 (30.36%)	1026 (75.61%)	331 (24.39%)			
	Rural	1850 (41.39%)	1466 (79.24%)	384 (20.76%)			
Marital status	Married and living with the spouse	2206 (49.35%)	1806 (81.87%)	400 (18.13%)	23.315	< 0.001	0.072
	Others	2264 (50.65%)	1720 (75.97%)	544 (24.03%)			
Economic status	Rich	1015 (22.71%)	913 (89.95%)	102 (10.05%)	199.270	< 0.001	0.211
	Common	3098 (69.30%)	2417 (78.02%)	681 (21.98%)			
	Poor	357 (7.99%)	196 (54.90%)	161 (45.10%)			
Health	Good	2344 (52.44%)	2119 (90.40%)	225 (9.60%)	534.868	< 0.001	0.346
	General	1644 (36.78%)	1182 (71.90%)	462 (28.10%)			
	Bad	482 (10.78%)	225 (46.68%)	257 (53.32%)			
Health changes	Better	648 (14.50%)	561 (86.57%)	87 (13.43%)	280.899	< 0.001	0.251
	Almost the same	2381 (53.26%)	2042 (85.76%)	339 (14.24%)			
	Worse	1441 (32.24%)	923 (64.05%)	518 (35.95%)			
Cognitive function	Cognitive impairment	43 (0.96%)	30 (69.77%)	13 (30.23%)	2.165	0.141	0.022
	Normal	4427 (99.04%)	3496 (78.97%)	931 (21.03%)			
Actual living arrangements	With children	1442 (32.26%)	1134 (78.64%)	308 (21.36%)	0.074	0.786	0.004
	Living alone or only with the spouse	3028 (67.74%)	2392 (79.00%)	636 (21.00%)			
Living arrangement preferences	With children	1572 (35.17%)	1175 (74.75%)	397 (25.25%)	24.898	< 0.001	0.075
	Living alone or only with the spouse	2898 (64.83%)	2351 (81.12%)	547 (18.88%)			
Fit between preferences and reality	Yes	3770 (84.34%)	3025 (80.24%)	745 (19.76%)	26.623	< 0.001	0.077
	No	700 (15.66%)	501 (71.57%)	199 (28.43%)			

### Living arrangements and depression

First, in model 1, we explored the relationship between different socio-demographic variables and actual living arrangements and depression in older adults. The results indicate that female and older adults with other marital statuses have a higher risk of depression (OR = 1.399, 1.564). The risk of depression among older adults living in town was 1.468 times that of city older adults. The risk of depression of older adults with common or poor economic status is higher than that of older adults with rich economic status (OR = 1.947, 3.614). Compared with older adults with good self-perceived health status, those with average and bad health status have a higher risk of depression (OR = 2.788, OR = 6.228). Compared to older adults who feel that their health has deteriorated in the past year, those who feel better or have little difference in self-perception have a lower risk of depression (OR = 0.484, OR = 0.493). At the same time, after controlling the role of covariates, older adults who actually live alone or only with the spouse are at a higher risk of depression, 1.261 times higher than those who live with children (*P* < 0.05). According to model 1, we further added the variable of living arrangement preferences to form model 2. The results showed that, under the control of other factors, the risk of depression of older adults who preferred living alone or only with their spouse was relatively low (OR = 0.553). At the same time, after controlling the living arrangement preferences, older adults who actually live alone or only with the spouse have a higher risk of depression than model 1. Finally, in Model 3, we include whether the living arrangement preferences are in line with reality in the model. We found that older adults who did not match their living arrangement preferences with reality had a relatively high risk of depression (OR = 1.320). At the same time, compared with model 2, the risk of depression of older people who actually live alone or only with the spouse decreases, and the risk of depression of older people who prefer living alone or only with spouse increases, after incorporating the variable of whether the living arrangement preferences conform to reality (Table 2). Meanwhile, considering the possible multicollinearity in logistic regression analysis, VIF was used to evaluate multicollinearity. The results showed that the VIF of all independent variables in this study was less than 10, indicating the absence of multicollinearity.

**Table 2:** Binary-logistic regression between living arrangements and depression

Variables		Model1			Model2			Model3		
		OR	95%CI	P	OR	95%CI	P	OR	95%CI	P
Age	One year increase	0.998	0.988–1.007	0.619	0.996	0.987–1.006	0.442	0.997	0.987–1.006	0.522
Gender	Male	Ref			Ref			Ref		
	Female	1.399	1.187–1.648	< 0.001	1.397	1.185–1.647	< 0.001	1.392	1.180–1.641	< 0.001
Residence	City	Ref			Ref			Ref		
	Town	1.468	1.192–1.807	< 0.001	1.442	1.171–1.777	0.001	1.422	1.153–1.752	0.001
	Rural	1.177	0.962–1.439	0.113	1.140	0.931–1.394	0.204	1.129	0.922–1.382	0.240
Marital status	Married and living with the spouse	Ref			Ref			Ref		
	Others	1.564	1.277–1.916	< 0.001	1.553	1.266–1.905	< 0.001	1.562	1.274–1.916	< 0.001
Economic status	Rich	Ref			Ref			Ref		
	Common	1.947	1.540–2.461	< 0.001	1.944	1.537–2.461	< 0.001	1.929	1.524–2.441	< 0.001
	Poor	3.614	2.622–4.982	< 0.001	3.540	2.564–4.889	< 0.001	3.506	2.538–4.843	< 0.001
Health	Good	Ref			Ref			Ref		
	General	2.788	2.320–3.351	< 0.001	2.785	2.316–3.349	< 0.001	2.779	2.310–3.342	< 0.001
	Bad	6.228	4.842–8.010	< 0.001	6.265	4.866–8.066	< 0.001	6.248	4.851–8.048	< 0.001
Health changes	Worse	Ref			Ref			Ref		
	Better	0.484	0.369–0.635	< 0.001	0.477	0.363–0.626	< 0.001	0.474	0.361–0.622	< 0.001
	Almost the same	0.493	0.413–0.589	< 0.001	0.491	0.411–0.586	< 0.001	0.489	0.409–0.585	< 0.001
Cognitive function	Cognitive impairment	Ref			Ref			Ref		
	Normal	0.820	0.397–1.693	0.591	0.857	0.413–1.776	0.677	0.858	0.413–1.785	0.682
Actual living arrangements	With children	Ref			Ref			Ref		
	Living alone or only with the spouse	1.261	1.024–1.553	0.029	1.838	1.430–2.363	< 0.001	1.732	1.351–2.221	< 0.001
Living arrangement preferences	With children	Ref			Ref			Ref		
	Living alone or only with the spouse				0.553	0.448–0.684	< 0.001	0.611	0.491–0.761	< 0.001
Fit between preferences and reality	Yes							Ref		
	No							1.320	1.060–1.643	0.013

**Fig. 2:**
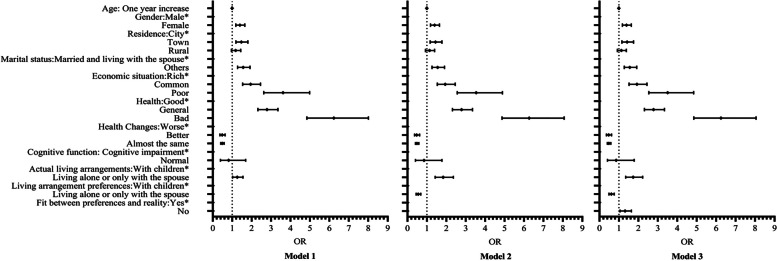
Forest map of binary logistic regression analysis results

We plotted the OR value corresponding to each variable in each model to more intuitively display the results of binary logical regression analysis, as shown in Fig. 2.

### Stability test

In order to ensure the reliability of the research results, we further utilized a multiple linear regression model to conduct regression analysis using the total depression score of older adults as the dependent variable and conducted stability tests on the main models (Models 1–3) of this study. Similar to the three models in binary logistic regression analysis (model1-3), multiple linear regression analysis is also divided into three models (model4-6). Firstly, in Model 4, we conducted regression analysis using the sociodemographic characteristics and actual living arrangements of older adults as independent variables (Corresponding to Model 1 in binary logistic regression analysis). Secondly, based on Model 4, living arrangement preference was added as an independent variable for regression analysis to form Model 5 (Corresponding to Model 2 in binary logistic regression analysis). Finally, in model 6, the fit between preference and reality was further added as an independent variable for regression analysis (Corresponding to Model 3 in binary logistic regression analysis). See supplementary files. The stability test indicates that our research findings on the relationship between living arrangements and depression in older adults are reliable.

## Discussion

This study selected the data of the latest wave (2017/2018) in the CLHLS. Since it can cover most of China, we believe that the related factors of depression reported based on this data can reflect the real situation of older adults in China. As far as we know, this is also the first article to consider the possible connection between actual living arrangements, living arrangement preferences, and the fit between preferences and reality with older adults’ depression. In this study, we first conducted a univariate analysis using the chi-square test. Then, in binary logistic regression analysis, the socio-demographic characteristics and actual living arrangements, living arrangement preferences, and the fit between preferences and reality are gradually included in Models 1 to 3 to explore the potential relationship between living arrangements related variables and depression when other factors are controlled. Finally, we conducted a stability test on the main conclusions of this study using multiple linear regression, and the results showed that the conclusion on the relationship between living arrangement related variables and depression in the older adults in this study is reliable.

### Actual living arrangements and depression

First of all, like other studies, we paid attention to the association between actual living arrangements and depression, which is also the field of most current studies. Through the binary-logistic regression analysis, we found that whether the socio-demographic variables were controlled in Model 1, or the potential impact of living arrangement preferences and the fit between preferences and reality was further controlled in Model 2 and Model 3, the actual living arrangements are significantly related to depression. In addition, older adults who actually live alone or only with the spouse have a higher risk of depression, compared with older adults living with children.

This result is not difficult to understand. Social support is necessary for living a happy and healthy life, and it is also of great significance for the well-being and health of older adults [43]. Many previous studies have identified a negative relevance between social support and older adults’ depression [44–46]. At the same time, the social convoy model also pointed out that social relationships are divided into three intimacy levels, which can be expressed by three concentric circles. The innermost circle indicates the closest social partner, usually including spouse and children. Compared with members in the outer circle, the interaction between the innermost people and older adults is the most frequent, and older adults get more support from members in the inner circle [29, 47, 48]. Another noteworthy point is that for Chinese people who emphasize the role of family and clan in traditional culture, intergenerational support from children may have different special meanings [49, 50]. In addition, the physical health status of older adults deteriorates with age. For older adults who have lost their ability to work and lack sources of income, the increasing demand for medical services is difficult to afford, so they have to rely on the care and financial support of their children [51].

### Living arrangement preferences and depression

The second is the potential relationship between living arrangement preferences and depression, which is an area of less concern in the current research and is also the key issue that this paper wants to explore. Based on binary-logistic regression analysis model 1, we added a variable formation model 2 of living arrangement preferences. Later, in model 3, we further discussed the potential relationship between living arrangement preferences and older adults’ depression under controlling whether preferences fit with reality. Whether in model 2 or model 3, we found that the living arrangement preferences are significantly related to older adults’ depression. The risk of depression in older adults who prefer to live alone or only with the spouse is relatively low, and the OR values are 0.553 and 0.611 respectively in model 2 and model 3. At the same time, after controlling the living arrangement preferences, the depression risk of older adults who actually live alone or only with the spouse rose from 1.261 in Model 1 to 1.838 in Model 2, indicating that the living arrangement preferences can not only significantly affect the depression level of older adults, but also a confounding factor of the actual living arrangements.

In older adults, the relationship between living arrangement preferences and depression is just opposite to that of actual living arrangements, which makes us very confused. Because living with children is a traditional cultural paradigm in Chinese traditional filial piety culture, it can be understood that older adults who actually live alone or only with their spouse have a relatively high risk of depression. However, it is difficult to explain why older adults who prefer living alone or only with the spouse have a low degree of depression, which is contrary to the traditional cultural paradigm, and the previous literature rarely involves this field.

We speculate that it may be correlated to the self-conditions of older adults. For older adults, not living with their children may mean a sense of freedom, as they are able to make independent decisions and actions without the need to consider and accommodate the habits of families composed of children [52]. However, as a relatively challenging choice, not all older adults will choose not to live with their children [53]. First of all, the higher a person's socio-economic status and good health in old age, the higher the possibility of living alone in old age [53, 54], and these conditions are often associated with lower depression [55, 56]. Secondly, successful aging means that they have confidence in their ability to live alone. They believe that they can live alone and adapt to the environment [53, 57, 58], and this confidence has also been proven to be an important factor that can related to the level of depression [59, 60].

### Fit between living arrangement preferences and reality and depression

Finally, we also explored the potential connection between the fit between the living arrangement preferences and reality with the depression of older adults. After controlling the confounding effects of actual living arrangements, living arrangement preferences, and socio-demographic characteristics, we found that the fit between preferences and reality is significantly related to the depression of older adults. Specifically, compared with older adults whose preferences fit with reality, the risk of depression of older adults who are not fit is 1.320 times higher (*P* < 0.05). This result is consistent with Chen's research [32]. Moreover, after controlling for the variable of whether the living arrangement preferences fit with reality, the risk of depression of older adults who actually live alone or only with spouse decreased from 1.838 to 1.732, and the risk of depression of older adults who prefer to live alone or only with spouse increased from 0.553 to 0.611, indicating that the fit between living arrangement preferences with the reality is not only a significant influencing factor for older adults’ depression but also a confounding factor of the living arrangements and the living arrangement preferences on depression.

The Strain Theory points out that the difference between desire and reality is one of the important causes of stress. The greater the difference between desire and reality, the greater the pressure [61]. This theory can also be applied to the care of older adults [62]. At the same time, according to discrepancy theories, the difference between the actual self and the ideal self is related to negative emotions [63]. In terms of living arrangements, the actual self represents the actual living arrangements, while the ideal self represents older adults' living arrangement preferences. The difference between preferences and reality may lead to negative emotions in older adults [64]. Chen and our research support the hypothesis of the discrepancy theories to some extent, that is, when the difference between individual preferences and reality is small, the probability of depression is lower [32]. On the other hand, the fit theory also provides theoretical support for our research. From the perspective of person-environment fit, it is believed that the fit between individual needs and environmental resources can affect individual happiness [65]. Chau et al. found that the improvement of person-environment fit is related to the reduction of depression scores in older adults [66]. From the perspective of expectation-satisfaction fit, some studies have shown that the fit between expectation and satisfaction will affect people's psychological well-being. When there are unrealistic and excessive demands, there will be a mismatch between expectations and satisfaction, which will lead to depression or anxiety, and other mental problems [67, 68].

### Application value and related suggestions

Under the premise of controlling other social demography characteristics, this study found that these three variables were significantly related to the depression of older adults by gradually adding actual living arrangements, living arrangement preferences, and whether the preference fit with reality in models 1–3. This is of great significance for further clarifying the relationship between older adults' living arrangements and depression and also provides inspiration for the formulation of elderly care policies and elderly care.

For family members who take care of older adults, we suggest that they consider various factors when considering their elders' living arrangements, especially respecting the individual wishes of older adults. For the formulation of elderly care policies, elderly security is an important social public issue, and building a safe, reliable, and comprehensive elderly social security system can help promote stable social and economic development. Firstly, it is necessary to improve the construction of the elderly security system, formulate corresponding policies for older adults with different living arrangements, strengthen support for vulnerable groups of older adults, ensure the basic living and spiritual needs of older adults, and build an elderly-friendly society. Secondly, it is necessary to promote and guide the correct concept of elderly care, break the stigma of "non-traditional living methods", and respect the living preferences of older adults. On the one hand, it is necessary to guide older adults to choose a suitable way of living based on their actual situation and wishes. On the other hand, it is necessary to effectively strengthen the care for empty nest elderly and solitary elderly, vigorously develop community elderly care, medical care integration, and other elderly care methods, promote on-site employment, maintain traditional family elderly care methods, and promote the compatibility between the actual living arrangements and wishes of older adults.

Another point that needs to be emphasized is that the variables related to living arrangements in this study are only one aspect of factors related to depression in older adults. We conducted targeted research on this issue because we found that current research has paid little attention to this issue. However, this does not mean that other factors have no impact on depression in older adults. At least, in this study, it was found that factors such as health and economic status can be significantly correlated with depression in older adults, and have a higher odds ratio.

## Limitations and future research

Finally, we remind future researchers of the limitations of this study. First, although the data selected in this study is from the CLHLS, a long-term longitudinal study, this study only focuses on the latest wave of cross-sectional data, and future researchers should take notice of the limitations of the cross-sectional study itself. Because cross-sectional studies cannot clearly determine or explain causal relationships. Subsequent researchers can conduct longitudinal studies based on this study to further determine the causal relationship between variables related to living arrangements and depression among older adults. Second, the CLHLS adopts the method of a questionnaire survey. Considering the differences in cultural backgrounds, older adults in China may have different expressions about living arrangements and depression from older adults in other countries. The researchers should consider the differences in cultural backgrounds when applying the conclusions. At the same time, the history of depression in older adults is also a factor that should be considered, and future researchers should consider its potential impact in collecting data. Although this study has some limitations, we still believe that it is valuable because it focuses on the potential relevance between older adults' living arrangements and depression, which supplements important literature in this field. Finally, we suggest that future researchers should attach importance to the impact of living arrangement preferences on older adults' mental health, which may be a complex mechanism.

## Conclusion

In a word, this paper explores the potential relationship between living arrangements and older adults' depression by constructing binary-logistic regression models. Our study found that the actual living arrangements, living arrangement preferences, and the fit between living arrangement preferences and reality are significantly related to older adults' depression levels. Specifically, older adults who actually live alone or only with the spouse have a higher risk of depression (OR values in models 1, 2, and 3 are 1.261, 1.838, and 1.732 respectively). Older adults who prefer living alone or only with the spouse have a relatively low risk of depression (OR values in model 2 and model 3 are 0.553 and 0.611 respectively). Older adults whose living arrangement preferences do not match reality are at higher risk of depression (OR = 1.320). We suggest that future researchers should not only be concerned about the influence of actual living arrangements on older adults but also take into account the personal preferences of older adults and the fit between preferences and reality when conducting research in this field.

### Supplementary Information

**Additional file 1:** **Table 1.** Multiple linear regression analysis of social demographic characteristics and actual living arrangements on depression in older adults (Model 4). **Table 2.** Multiple linear regression analysis of social demographic characteristics, actual living arrangements, and living arrangement preferences on depression in older adults (Model 5). **Table 3.** Multiple linear regression analysis of social demographic characteristics, actual living arrangements, living arrangement preferences, and fit between preferences and reality on depression in older adults (Model 6).

## Data Availability

The dataset supporting the conclusions of this article is available in the [Peking University Open Research Data Platform] repository, [unique persistent identifier and hyperlink to dataset in https://opendata.pku.edu.cn/dataset.xhtml?persistentId=doi:10.18170/DVN/WBO7LK].

## Abbreviations

CLHLS: Chinese Longitudinal Healthy Longevity Survey
CMMSE: The Chinese version of the Mini-Mental State Examination
CES-D-10: 10-Item Center for Epidemiologic Studies Depression Scale
OR: Odds ratio
95% CI: 95% Confidence interval
