# Supplementary material for: Living arrangements and depression of the older adults– evidence from the Chinese longitudinal healthy longevity survey
Source: BMC Public Health. 2023 Sep 27;23:1870. doi: 10.1186/s12889-023-16730-4 (PMC10523833; doi:10.1186/s12889-023-16730-4)
Supplement: Supplementary file 1 — Additional file 1: Table 1. Multiple linear regression analysis of social demographic characteristics and actual living arrangements on depression in older adults (Model 4). Table 2. Multiple linear regression analysis of social demographic characteristics, actual living arrangements, and living arrangement preferences on depression in older adults (Model 5). Table 3. Multiple linear regression analysis of social demographic characteristics, actual living arrangements, living arrangement preferences, and fit between preferences and reality on depression in older adults (Model 6). [file 12889_2023_16730_MOESM1_ESM.docx]

| Variables | | B | S.E | β | t | p | VIF |
| --- | --- | --- | --- | --- | --- | --- | --- |
| Age | One year increase | 0.003 | 0.006 | 0.007 | 0.480 | 0.631 | 1.295 |
| Gender | Male | Ref | | | | | |
|  | Female | 0.499 | 0.113 | 0.060 | 4.410 | ＜0.001 | 1.097 |
| Residence | City | Ref | | | | | |
|  | Town | 0.529 | 0.142 | 0.059 | 3.717 | ＜0.001 | 1.470 |
|  | Rural | 0.316 | 0.133 | 0.038 | 2.368 | 0.018 | 1.480 |
| Marital status | Married and living with the spouse | Ref | | | | | |
|  | Others | 0.636 | 0.143 | 0.077 | 4.436 | ＜0.001 | 1.763 |
| Economic status | Rich | Ref | | | | | |
|  | Common | 1.078 | 0.133 | 0.120 | 8.104 | ＜0.001 | 1.291 |
|  | Poor | 2.547 | 0.232 | 0.167 | 10.969 | ＜0.001 | 1.360 |
| Health | Good | Ref | | | | | |
|  | General | 1.892 | 0.122 | 0.221 | 15.475 | ＜0.001 | 1.194 |
|  | Bad | 4.125 | 0.200 | 0.310 | 20.601 | ＜0.001 | 1.325 |
| Health changes | Worse | Ref | | | | | |
|  | Better | -1.242 | 0.179 | -0.106 | -6.923 | ＜0.001 | 1.370 |
|  | Almost the same | -1.288 | 0.131 | -0.156 | -9.831 | ＜0.001 | 1.468 |
| Cognitive function | Cognitive impairment | Ref | | | | | |
|  | Normal | -0.152 | 0.560 | -0.004 | -0.272 | 0.786 | 1.024 |
| Actual living arrangements | With children | Ref | | | | | |
|  | Living alone or only with the spouse | 0.354 | 0.148 | 0.040 | 2.386 | 0.017 | 1.652 |
| R | 0.490 | | | | | | |
| R2 | 0.240 | | | | | | |
| Adjusted R2 | 0.238 | | | | | | |
| F | 108.45*** | | | | | | |

Table1 Multiple linear regression analysis of social demographic characteristics and actual living arrangements on depression in older adults (Model 4)

Table2 Multiple linear regression analysis of social demographic characteristics, actual living arrangements, and living arrangement preferences on depression in older adults (Model 5)

| Variables | | B | S.E | β | t | p | VIF |
| --- | --- | --- | --- | --- | --- | --- | --- |
| Age | One year increase | 0.002 | 0.006 | 0.004 | 0.248 | 0.804 | 1.299 |
| Gender | Male | Ref | | | | | |
|  | Female | 0.497 | 0.113 | 0.060 | 4.398 | ＜0.001 | 1.097 |
| Residence | City | Ref | | | | | |
|  | Town | 0.521 | 0.142 | 0.058 | 3.665 | ＜0.001 | 1.470 |
|  | Rural | 0.288 | 0.133 | 0.034 | 2.162 | 0.031 | 1.483 |
| Marital status | Married and living with the spouse | Ref | | | | | |
|  | Others | 0.612 | 0.143 | 0.074 | 4.276 | ＜0.001 | 1.765 |
| Economic status | Rich | Ref | | | | | |
|  | Common | 1.071 | 0.133 | 0.120 | 8.072 | ＜0.001 | 1.292 |
|  | Poor | 2.511 | 0.232 | 0.165 | 10.829 | ＜0.001 | 1.362 |
| Health | Good | Ref | | | | | |
|  | General | 1.887 | 0.122 | 0.220 | 15.460 | ＜0.001 | 1.194 |
|  | Bad | 4.114 | 0.200 | 0.309 | 20.581 | ＜0.001 | 1.325 |
| Health changes | Worse | Ref | | | | | |
|  | Better | -1.243 | 0.179 | -0.106 | -6.943 | ＜0.001 | 1.370 |
|  | Almost the same | -1.285 | 0.131 | -0.155 | -9.828 | ＜0.001 | 1.468 |
| Cognitive function | Cognitive impairment | Ref | | | | | |
|  | Normal | -0.106 | 0.558 | -0.003 | -0.190 | 0.849 | 1.025 |
| Actual living arrangements | With children | Ref | | | | | |
|  | Living alone or only with the spouse | 0.759 | 0.175 | 0.086 | 4.339 | ＜0.001 | 2.305 |
| Living arrangement preferences | With children | Ref | | | | | |
|  | Living alone or only with the spouse | -0.650 | 0.150 | -0.075 | -4.348 | ＜0.001 | 1.758 |
| R | 0.494 | | | | | | |
| R2 | 0.244 | | | | | | |
| Adjusted R2 | 0.241 | | | | | | |
| F | 102.459*** | | | | | | |

Table3 Multiple linear regression analysis of social demographic characteristics, actual living arrangements, living arrangement preferences, and fit between preferences and reality on depression in older adults (Model 6)

| Variables | | B | S.E | β | t | p | VIF |
| --- | --- | --- | --- | --- | --- | --- | --- |
| Age | One year increase | 0.003 | 0.006 | 0.007 | 0.453 | 0.651 | 1.303 |
| Gender | Male | Ref | | | | | |
|  | Female | 0.488 | 0.113 | 0.059 | 4.325 | ＜0.001 | 1.097 |
| Residence | City | Ref | | | | | |
|  | Town | 0.491 | 0.142 | 0.055 | 3.454 | 0.001 | 1.476 |
|  | Rural | 0.265 | 0.133 | 0.032 | 1.992 | 0.046 | 1.487 |
| Marital status | Married and living with the spouse | Ref | | | | | |
|  | Others | 0.624 | 0.143 | 0.075 | 4.363 | ＜0.001 | 1.766 |
| Economic status | Rich | Ref | | | | | |
|  | Common | 1.061 | 0.133 | 0.118 | 7.999 | ＜0.001 | 1.292 |
|  | Poor | 2.483 | 0.232 | 0.163 | 10.717 | ＜0.001 | 1.364 |
| Health | Good | Ref | | | | | |
|  | General | 1.879 | 0.122 | 0.219 | 15.409 | ＜0.001 | 1.194 |
|  | Bad | 4.101 | 0.200 | 0.308 | 20.541 | ＜0.001 | 1.326 |
| Health changes | Worse | Ref | | | | | |
|  | Better | -1.257 | 0.179 | -0.107 | -7.026 | ＜0.001 | 1.371 |
|  | Almost the same | -1.288 | 0.131 | -0.155 | -9.861 | ＜0.001 | 1.468 |
| Cognitive function | Cognitive impairment | Ref | | | | | |
|  | Normal | -0.112 | 0.558 | -0.003 | -0.201 | 0.841 | 1.025 |
| Actual living arrangements | With children | Ref | | | | | |
|  | Living alone or only with the spouse | 0.729 | 0.175 | 0.083 | 4.170 | ＜0.001 | 2.311 |
| Living arrangement preferences | With children | Ref | | | | | |
|  | Living alone or only with the spouse | -0.533 | 0.153 | -0.062 | -3.481 | 0.001 | 1.849 |
| Fit between preferences and reality | Yes | Ref | | | | | |
|  | No | 0.527 | 0.153 | 0.046 | 3.441 | 0.001 | 1.072 |
| R | 0.496 | | | | | | |
| R2 | 0.246 | | | | | | |
| Adjusted R2 | 0.243 | | | | | | |
| F | 96.650*** | | | | | | |
